# Types and Rates of COVID-19 Vaccination in Patients With Newly Diagnosed Microsatellite Stable and Instable Non-Metastatic Colon Cancer

**DOI:** 10.7759/cureus.61780

**Published:** 2024-06-06

**Authors:** Erman Akkus, Bahar Karaoglan, Cihangir Akyol, Ali Ekrem Ünal, Mehmet Ayhan Kuzu, Berna Savaş, Güngör Utkan

**Affiliations:** 1 Department of Medical Oncology, Ankara University School of Medicine, Ankara, TUR; 2 Department of General Surgery, Ankara University School of Medicine, Ankara, TUR; 3 Department of Surgical Oncology, Ankara University School of Medicine, Ankara, TUR; 4 Department of Pathology, Ankara University School of Medicine, Ankara, TUR

**Keywords:** microsatellite instable colon cancer, dmmr, colon cancer, bnt162b2, covid-19 vaccine

## Abstract

Introduction

Microsatellite instable (deficient mismatch repair, dMMR) colon cancer is associated with hypermutability and immune infiltration-activation. COVID-19 vaccines stimulate immune-inflammation response. This study aimed to investigate the types and rates of COVID-19 vaccines in patients with newly diagnosed colon cancer and compare it according to the microsatellite status.

Methods

The study was a single-center case-control study. Patients diagnosed with colon cancer at least three months after the last COVID-19 vaccine (BNT162b2, CoronaVac) dose were included. Patients with dMMR and microsatellite stable (MSS) tumors were defined as cases and controls, respectively, between June 2021 and June 2023. Baseline characteristics and vaccine status between case-control groups were compared as univariable and multivariable. Inflammation markers were compared between MSS+CoronaVac and dMMR+BNT162b2 groups.

Results

A total of 76 patients were included. The BMI was higher in the MSS group (BMI>25 84.3% vs. 57.9%, p=0.00), and right-sided tumors were more common in the dMMR group (71% vs.46.4%, p=0.00). The dMMR group had a higher BNT162b2 vaccine history than the MSS group (86.8% vs. 63.2%, p=0.01), while there was no difference in CoronaVac history (p=0.32). Significant variables in univariable analysis (BMI, localization, and BNT162b2) were included in multivariable logistic regression. The BNT162b2 vaccine was significantly associated with dMMR status (OR: 6.39, 95% CI: 1.55-26.26, p=0.01). The dMMR+BNT162b2 group had higher median C-reactive protein (CRP) level (p=0.01), erythrocyte sedimentation rate (p=0.05), and lower lymphocyte/CRP ratio (p=0.04) than the MSS+CoronaVac group.

Conclusion

Immune infiltration in dMMR colon cancer may interact with COVID-19 vaccine-induced immune activation. Long-term clinical and preclinical studies are needed to confirm these findings.

## Introduction

Colorectal cancer (CRC) is the third most common cancer in males and second in females, with approximately one million deaths per year worldwide [1]. Hereditary and modifiable risk factors have a role in the occurrence of CRC, such as hereditary nonpolyposis colorectal cancer (HNPCC) as a hereditary risk factor, and obesity, diabetes, and smoking as modifiable risk factors [2].

Four consensus molecular subtypes of CRC have been described [3]. First subtype is the “MSI immune,” which is characterized by microsatellite instability (MSI) and strong immune activation-infiltration. Microsatellites are the regions of repeated DNA sequences. MSI results from misfunctioning of the DNA mismatch repair (MMR) system. Thus, deficient MMR (dMMR) is a marker of genetic hypermutability, which causes malignant transformation. The dMMR phenotype of CRC is a distinct entity from microsatellite stable (MSS) ones, which shows different clinical and pathological features such as poor differentiation, prominent lymphocytic infiltration, and right-sided colon location. MSI testing is recommended for all CRC patients to determine prognosis and guide treatment decisions. MSI testing helps in deciding adjuvant chemotherapy in a non-metastatic setting and also for first-line immunotherapy in a metastatic setting [4].

The mRNA-based COVID-19 vaccine of Pfizer-BioNTech (BNT162b2, tozinameran) was one of the most used vaccines during the pandemic. The vaccine delivers mRNAs (coding spike protein of the virus) into the cells of the body, makes them express the protein, and, finally, stimulates immune response and inflammation [5,6]. It has been shown that PD-L1 surface expression on peripheral blood granulocytes and monocytes increases after mRNA COVID-19 vaccine [7]. Analyzing the systems biology effects of COVID-19 mRNA vaccines has shown that BNT162b2 vaccine affects transcription of immune response, inflammation, cell adhesion, and proliferation pathways, and this effect is similar to those seen with protein synthesis inhibitors, ATPase inhibitors, topoisomerase inhibitors, DNA synthesis inhibitors, and mitomycin-c [8]. There is a concern that mRNA-based vaccines may cause translation of undesired proteins [9]. It has been shown that the mRNA in the vaccine is reverse transcribed intracellularly into DNA in vitro liver cells [10]. Moreover, mRNA-based vaccine causes increased DNA damage in blood mononuclear cells of healthy subjects, partly due to increased oxidative stress [11]. Interestingly, inflammatory stimuli may cause alterations in the MMR system [12].

dMMR is related to hypermutability and immune infiltration-activation. The mRNA-based COVID-19 vaccine stimulates immune-inflammation response and has probable intracellular genomic and hemostasis effects. Thus, mRNA-based COVID-19 vaccine may be associated with clinically symptomatic detection of dMMR CRC via immune activation in the tumor. The aim of this case-control study is to investigate the association between mRNA-based COVID-19 vaccine and newly diagnosed CRC with dMMR.

## Materials and methods

Patients and study design

The study was designed as a single-center, retrospective, case-control study. Cases were defined as tumors with dMMR and controls were defined as tumors with MSS. Inclusion criteria were as follows: any gender, age >18 years, histology-confirmed, newly diagnosed non-metastatic CRC patients, IHC-confirmed dMMR (for cases) and MSS (for controls) in tumor, CoronaVac or Pfizer-BioNTech (Tozinameran, BNT162b2) vaccination for COVID-19, available vaccine data, at least three months’ interval from the last vaccine dose to the diagnose of CRC, and available clinical and pathologic data. Patients without planned data were excluded. The sample size was calculated using Epi Info™ as an unmatched case-control study. A two-sided confidence level of 95%, power of 80%, ratio of controls to cases 1, percent of controls exposed 50%, and the expected odd ratio of 5 were used for the calculation. Kelsey, Fleiss, and Fleiss with continuity correction methods revealed 32, 31, and 36 patients per group, respectively.

**Figure 1:**
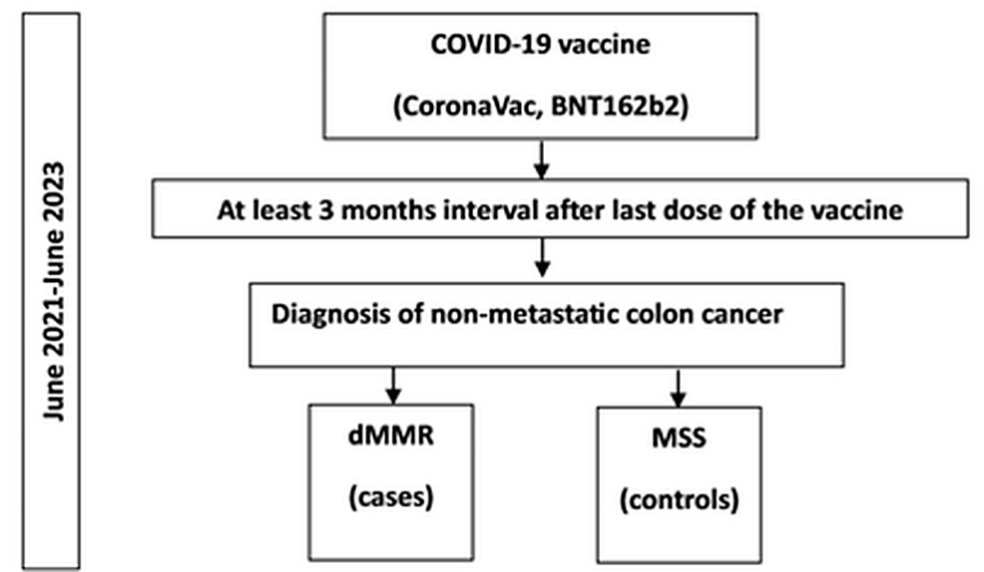
Study design dMMR, deficient mismatch repair; MSS, microsatellite stable

The CoronaVac (Sinovac) vaccine was first used on January 13, 2021, in Turkey, and the date of first use of Pfizer-BioNTech vaccine (Tozinameran, BNT162b2) was April 12, 2021. Patients diagnosed with non-metastatic CRC between June 2021 and June 2023 at the Department of Medical Oncology, Ankara University Faculty of Medicine, Ankara, Turkey, were screened according to the inclusion criteria. In total, 38 cases (dMMR) were detected and included as the case group. Among 242 patients with MSS tumors, 38 patients were selected by simple random sampling and included as the control group (Figure 1).

The demographic, clinical, and pathologic data of the patients were recorded. Age, gender, body mass index (BMI), smoking history, diabetes mellitus, primary tumor localization (right-sided and left-sided), pathologic T (pT), pathologic N (pN), stage (according to the American Joint Committee on Cancer [AJCC] 8th Edition, 2017) and grade data (WHO grading) were determined. Vaccine type and dose were recorded. Leukocytes, neutrophils, lymphocytes, neutrophile/lymphocyte ratio (NLR), C-reactive protein (CRP), and erythrocyte sedimentation rate at the time of diagnosis were recorded.

Baseline characteristics and vaccine status between case and control groups were compared. Statistically significant variables were analyzed by multivariable analysis. Complete blood count (CBC) values (leukocytes, neutrophils, lymphocytes), NLR, CRP, erythrocyte sedimentation rate, and lymphocyte/CRP ratio values at the time of diagnosis were compared between the MSS+only CoronaVac and dMMR+only BNT162b2 groups.

Ethical approval was obtained from the Clinical Research Ethics Committee of Ankara University Faculty of Medicine (Number: 2022000659, 2022/659) in compliance with the Declaration of Helsinki.

Immunohistochemistry and CBC-biochemistry analysis

The Pathology Department analyzed MMR proteins of MSH2, MSH6, PMS2, and MLH1 in paraffin blocks of tumors using Optiview DAB, Ultraview Universal DAB, Ultraview AP Red immunohistochemistry kits, and Ventana Benchmark XT or Ultra systems. The CBC and biochemistry analysis was performed using the Abbott CELL-DYN Ruby Analyzer and Abbott Architect chemistry system. The reference values were evaluated according to institutional reference rates.

Statistical analysis

Continuous variables were presented as median (mini-mum (min)-maximum (max)) and mean (SD). Categorical variables were presented as percentage. Univariate analyses were performed using the chi-square test, Fisher exact test, Student’s t-test, and Mann-Whitney U test, where needed. The statistically significant variables in univariable analysis were included in the multivariable analysis. Multivariable analysis was performed by logistic regression analysis. All p-values were based on a two-tailed test of significance (p=0.05). All the statistical analyses were conducted using SPSS Version 26 (IBM Corp., Armonk, NY).

## Results

Baseline characteristics

A total of 76 patients (38 per group) were included in the study according to the inclusion and exclusion criteria. The flow diagram of the selection of the study population is presented in Figure 1. The characteristics of the case (dMMR) and control (MSS) groups are presented in Table 1. The mean (SD) age of the patients was 62.5 (11.3) and 63.8 (13.6) years in the MSS and dMMR groups, respectively, and 55.3% of the patients were male in both groups. Smoking and diabetes history did not differ between groups. However, 84.3% of the patients in the MSS group had a BMI above 25, whereas 57.9% in the dMMR group (p=0.00). Also, 71% of the tumors were right-sided in the dMMR group compared to 46.4% in the MSS group (p=0.00). pT, pN, stage, and grade parameters were not different between MSS and dMMR patients.

**Table 1:** Characteristics of MSS and dMMR groups MSS, microsatellite stable; dMMR, deficient mismatch repair

	MSS (n=38)	dMMR (n=38)	p-Value
Age (mean, SD)	62.5 (11.3)	63.8 (13.6)	0.63
Gender (n, %)			
Male	21 (55.3)	21 (55.3)	1
Female	17 (44.7)	17 (44.7)	
BMI, kg/m^2 ^(n, %)			
<25	6 (15.7)	16 (42.1)	0.00
>25	32 (84.3)	22 (57.9)	
Smoking (n, %)			
Never	18 (47.3)	16 (42.1)	0.87
Ex-smoker	16 (42.1)	17 (44.7)	
Current smoker	4 (10.6)	5 (13.2)	
Diabetes mellitus (n, %)			
Present	10 (26.3)	14 (36.8)	0.32
Absent	28 (73.7)	24 (63.2)	
Tumor localization (n, %)			
Right-sided	13 (46.4)	27 (71)	0.00
Left-sided	25 (53.6)	11 (29)	
pT (n, %)			
T1	1 (2.6)	0 (0)	0.22
T2	3 (7.8)	1 (2.6)	
T3	29 (76.3)	26 (68.4)	
T4	5 (13.3)	11 (29)	
pN (n, %)			
N0	20 (52.7)	24 (63.2)	0.41
N1	12 (31.6)	7 (18.4)	
N2	6 (15.7)	7 (18.4)	
Stage (n, %)			
I	4 (10.6)	0 (0)	0.09
II	16 (42.1)	22 (57.9)	
III	18 (47.3)	16 (42.1)	
Grade (n, %)			
1	1 (2.6)	1 (2.6)	0.37
2	32 (84.2)	31 (81.6)	
3	5 (13.2)	6 (15.8)	

Vaccine and dMMR

**Figure 2:**
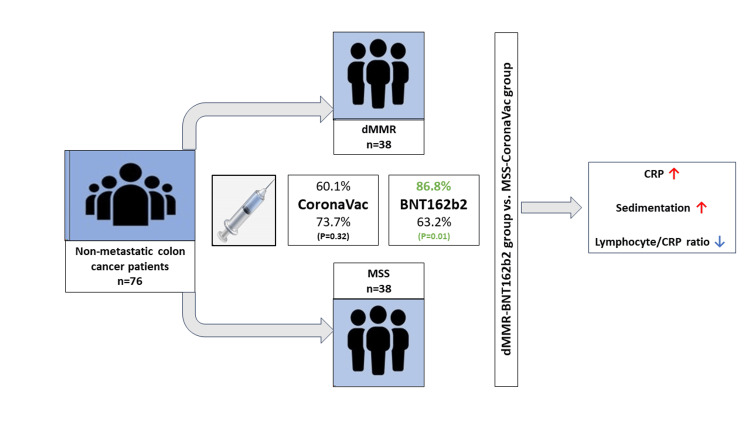
Association between COVID-19 vaccines and microsatellite instability status in non-metastatic colon cancer MSS, microsatellite stable; dMMR, deficient mismatch repair; CRP, C-reactive protein

The vaccine status of MSS and dMMR groups is presented in Table 2. Overall, 63.2% of the patients (n=24) in the MSS group and 86.8% of the patients (n=33) in the dMMR group had BNT162b2 vaccine history (p=0.01) (Figure 2). Of those, 81.9% had two or more doses of BNT162b2 in the dMMR group. Also, 73.7% of the patients (n=28) in the MSS group and 60.1% of the patients (n=23) in the dMMR group had CoronaVAc vaccine history (p=0.32). All patients had received two or more doses of the vaccine. The BNT162b2 vaccine was significantly more common in dMMR patients than in MSS patients, while CoronoVac did not differ between groups.

**Table 2:** Vaccine status of MSS and dMMR groups MSS, microsatellite stable; dMMR, deficient mismatch repair

	MSS (n=38)	dMMR (n=38)	p-Value
BNT162b2 (n, %)			
Yes	24 (63.2)	33 (86.8)	0.01
No	14 (36.8)	5 (13.2)	
BNT162b2 dose (n, %)			
1 dose	6 (25)	6 (18.1)	
2 doses	13 (54.1)	19 (57.6)	
3 doses	5 (20.9)	6 (18.1)	
4 doses	0	2 (6.2)	
CoronaVac (Sinovac) (n, %)			
Yes	28 (73.7)	23 (60.1)	0.32
No	10 (26.3)	15 (39.9)	
CoronaVac dose (n, %)			
2 doses	19 (67.9)	19 (82.6)	
3 doses	9 (32.1)	4 (17.4)	

The variables that were statistically significant in univariable analysis (BMI, tumor localization, and BNT162b2 vaccine) were included in the multivariable logistic regression analysis for MSI status (Table 3). The analysis revealed a significant model (chi-square=23.934, df=3, p=0.000). All variables were still statistically significant in multivariable analysis. The BNT162b2 vaccine was significantly associated with dMMR status (OR: 6.39, 95% CI: 1.55-26.26, p=0.01).

**Table 3:** Multivariable logistic regression analysis for MSI status* *Significant model (chi-square=23.934, df=3, p=0.000), constant; Exp(B):0.06, p=0.00 +Variables that were statistically significant in univariable analysis were included.

Variable^+^	OR (95% CI)	p-Value
BMI (<25 vs >25)	4.15 (1.18-14.65)	0.02
Localization (Right-sided vs left-sided)	5.07 (1.68-15.29)	0.00
BNT162b2 vaccine	6.39 (1.55-26.26)	0.01

Inflammation markers, vaccine, and dMMR

Since dMMR and mRNA-based COVID-19 vaccine are both associated with immune activation and infiltration, we evaluated inflammation markers. To see the difference, if any, we compared the MSS+only CoronaVac and dMMR+only BNT162b2 groups. The inflammation markers and ratios of the groups are presented in Table 4. Leukocytes, neutrophils, lymphocytes, and neutrophile/lymphocyte ratio were not different between groups. However, the dMMR+BNT162b2 group had a higher median CRP level than the MSS+CoronaVac group (p=0.01). Median sedimentation was higher in the dMMR+BNT162b2 group with a borderline p value (p=0.05). Lymphocyte/CRP ratio was significantly lower in the dMMR+BNT162b2 group (p=0.04).

**Table 4:** Inflammatory markers of MSS-CoronaVac and dMMR-BNT162b2 groups MSS, microsatellite stable; dMMR, deficient mismatch repair; NLR, neutrophile/lymphocyte ratio; CRP, C-reactive protein

	MSS+CoronaVac (n=14)	dMMR+BNT162b2 (n=15)	p-Value
Leukocyte, x10^3^/mcL (median, min-max)	7.95 (6.5-9.4)	7.9 (4.8-12.3)	0.76
Neutrophil, x10^3^/mcL (median, min-max)	4.9 (4.1-7)	4.9 (2.7-7.9)	0.97
Lymphocyte, x10^3^/mcL (median, min-max)	1.9 (0.6-2.9)	2 (1.3-3.2)	0.57
NLR (median, min-max)	2.37 (1.7-10.6)	2.37 (1.19-5.26)	0.61
CRP, mg/L (median, min-max)	6.6 (0.5-16.7)	14 (5.3-43)	0.01
Sedimentation, mm/h (median, min-max)	10 (3-35)	17.5 (4-60)	0.05
Lymphocyte/CRP ratio (median, min-max)	0.3 (0.09-5.8)	0.17 (0.03-0.37)	0.04

## Discussion

In this case-control study, we revealed that the mRNA-based COVID-19 vaccine is associated with dMMR non-metastatic colon cancer. The BNT162b2 vaccine is associated with the higher risk of dMMR non-metastatic colon cancer, while CoronaVac is not associated with MSI status. Moreover, patients who received the BNT162b2 vaccine and have dMMR colon cancer had higher levels of CRP and sedimentation and lower levels of lymphocyte/CRP ratio, which suggest an inflammatory and immune relationship between mRNA-based vaccine and dMMR status. The relationship between lower BMI, right-sided localization, and dMMR status was consistent with the literature.

The first question that emerged is the mechanism of the relationship between the mRNA-based vaccine and colon cancer with dMMR. Colon cancer is a multistep process from adenoma to malignancy, which almost takes approximately 10-15 years to develop [13]. Although we included patients with a history of vaccines before the diagnosis of colon cancer, the time interval (three months) is short and probably the patients already had the malignant lesion at the time of the vaccine. Thus, the most reasonable hypothesis is that the immune reactivation caused by the mRNA-based vaccine may make tumor symptomatic, leading to diagnosis because of the vaccine-induced inflammation. On the other hand, it has been previously shown that the mRNA-based BNT162b2 vaccine caused increased DNA damage in blood mononuclear cells of healthy subjects, partly due to increased oxidative stress [11]. The damage was transient and DNA repair capacity was intact. Therefore, another less reasonable hypothesis would be that the mRNA-based vaccine may cause damage to the MMR system of the tumor cells.

The dMMR (MSI-H) phenotype of CRC is distinct from MSS, exhibiting unique clinical and pathological characteristics. These include significant lymphocytic infiltration, which comprises activated cytotoxic T lymphocytes, Th1 cells, CD4+ T cells, NK cells, and macrophages [3]. The tumors with dMMR also have higher levels of interleukin (IL)-1, IL-6, interferon gamma (IFN-γ), and tumor necrosis factor [14]. IL-6 overexpression in colon cancer is pro-tumorigenic by promoting defects in the DNA MMR system [15]. PD-L1 expression and tumor mutation burden is much higher in the tumor microenvironment of tumors with dMMR than with MSS [16]. Therefore, the immune microenvironment of colon cancers with dMMR may make it prone to interaction with mRNA-based vaccine-induced inflammation. Clinically, it has been found that dMMR status in CRC was associated with increased neutrophil counts, and higher NLR and CRP levels in the early stage [17]. Consistently, CRP level was higher in the dMMR group in our study. NLR was not found to be associated with MSI status, consistent with our study [18]. Lymphocyte/CRP ratio was previously found to be prognostic in CRC [19]. Patients with the dMMR tumor had a lower lymphocyte/CRP ratio in our study.

The mRNA-based COVID-19 vaccine delivers mRNAs (coding spike protein of the virus) into the cells of the body, makes them express the protein, and, finally, stimulates immune response [5,6]. The BNT162b2 vaccine strikingly stimulates IFN-γ secretion from NK cells and CD8+ T cells. The CD8+ T cell response induced by BNT162b2 is dependent on type I interferon [20]. Although anti-COVID immunity weakens months after the mRNA-based vaccine, long-term effects on innate immunity have been shown, including higher IL-1/IL-6 release and decreased production of IFN-α [21]. These immune mechanisms may interact with the tumor microenvironment of colon cancer with dMMR. In a study including healthy subjects, the mRNA-based BNT162b2 vaccine caused increased DNA damage in blood mononuclear cells but did not influence the DNA repair capacity of PBMCs, showing that the vaccine successfully triggers the DNA damage response network [22]. However, the tumors with dMMR may be prone to the DNA damage effect of the vaccine. Despite the limited data, the mRNA-based COVID-19 vaccine may have intracellular effects. It has been shown that the mRNA in the vaccine was reverse transcribed intracellularly into DNA in vitro liver cells [10]. Analyzing the systems biology effects of COVID-19 mRNA vaccines has shown that the BNT162b2 vaccine affects transcription of immune response, inflammation, cell adhesion, and proliferation pathways, and this effect is similar to those seen with protein synthesis inhibitors, ATPase inhibitors, topoisomerase inhibitors, DNA synthesis inhibitors, and mitomycin-c [8]. With these data emerge the possibility of intracellular effects of mRNA-based COVID-19 vaccine in tumor cells.

There are some limitations of the study. Although the sample size was consistent with power analysis, the confirmation of these findings in larger samples would be informative. It may be still early to detect long-term effects of mRNA-based vaccines. However, a possible explanatory hypothesis is not that the vaccine causes dMMR, rather it may make the tumor active to diagnose. The study has the disadvantages due to its retrospective nature. Since this is a case-control study, it should not be interpreted as a cause-and-effect relationship. In vitro and in vivo preclinical studies are warranted to test the hypotheses we proposed and discussed here. Another limitation of our study is that we do not have the data of germline testing for HNCPP. However, the mean age of our study population was 63.8 years, which is higher than the mean age of HNCPP cases (45 years), suggesting that our cases are mainly sporadic cases. Finally, this study should not be interpreted and misused as a pitfall of the mRNA COVID-19 vaccine, which saved millions of lives during the devastating pandemic.

## Conclusions

This case-control study shows that the mRNA-based COVID-19 vaccine rate is higher in patients with microsatellite instable (dMMR) non-metastatic colon cancer. Immune-inflammatory mechanism may have a role in this association, which may be derived from facilitating the diagnosis of already existing tumor by immune-inflammatory interaction. Long-term clinical and preclinical studies are needed to confirm the findings of this hypothesis-generating study.
